# Perlecan domain 1 recombinant proteoglycan augments BMP-2 activity and osteogenesis

**DOI:** 10.1186/1472-6750-12-60

**Published:** 2012-09-11

**Authors:** Arthur A DeCarlo, Maria Belousova, April L Ellis, Donald Petersen, Hernan Grenett, Patrick Hardigan, Robert O’Grady, Megan Lord, John M Whitelock

**Affiliations:** 1 Agenta Biotechnologies, Inc, 1500 1st Ave. N., Unit 31, Birmingham, AL, 35203, USA; 2 Graduate School of Biomedical Engineering, University of New South Wales, Sydney, Australia; 3 Statistical Consulting Center, Nova Southeastern University, 3200 South University Dr, Ft. Lauderdale, FL, 33328, USA

**Keywords:** Osteogenesis, BMP-2, Heparan sulfate, Chondroitin sulfate, Proteoglycan, TCP, Bone graft, Implant, Osteoblast, Perlecan

## Abstract

**Background:**

Many growth factors, such as bone morphogenetic protein (BMP)-2, have been shown to interact with polymers of sulfated disacharrides known as heparan sulfate (HS) glycosaminoglycans (GAGs), which are found on matrix and cell-surface proteoglycans throughout the body. HS GAGs, and some more highly sulfated forms of chondroitin sulfate (CS), regulate cell function by serving as co-factors, or co-receptors, in GF interactions with their receptors, and HS or CS GAGs have been shown to be necessary for inducing signaling and GF activity, even in the osteogenic lineage. Unlike recombinant proteins, however, HS and CS GAGs are quite heterogenous due, in large part, to post-translational addition, then removal, of sulfate groups to various positions along the GAG polymer. We have, therefore, investigated whether it would be feasible to deliver a DNA pro-drug to generate a soluble HS/CS proteoglycan *in situ* that would augment the activity of growth-factors, including BMP-2, *in vivo*.

**Results:**

Utilizing a purified recombinant human perlecan domain 1 (rhPln.D1) expressed from HEK 293 cells with HS and CS GAGs, tight binding and dose-enhancement of rhBMP-2 activity was demonstrated *in vitro*. *In vitro*, the expressed rhPln.D1 was characterized by modification with sulfated HS and CS GAGs. Dose-enhancement of rhBMP-2 by a *pln.D1* expression plasmid delivered together as a lyophilized single-phase on a particulate tricalcium phosphate scaffold for 6 or more weeks generated up to 9 fold more bone volume *de novo* on the maxillary ridge in a rat model than in control sites without the *pln.D1* plasmid. Using a significantly lower BMP-2 dose, this combination provided more than 5 times as much maxillary ridge augmentation and greater density than rhBMP-2 delivered on a collagen sponge (InFuse™).

**Conclusions:**

A recombinant HS/CS PG interacted strongly and functionally with BMP-2 in binding and cell-based assays, and, *in vivo*, the *pln.247* expression plasmid significantly improved the dose-effectiveness of BMP-2 osteogenic activity for *in vivo de novo* bone generation when delivered together on a scaffold as a single-phase. The use of HS/CS PGs may be useful to augment GF therapeutics, and a plasmid-based approach has been shown here to be highly effective.

## Background

Commercial augmentation of synthetic bone graft substitutes began with approval of rhBMP-7 delivered in a putty of bovine type 1 collagen and carboxymethyl cellulose (OP-1™) under a Humanitarian Device Exemption by the FDA. This product was followed by the addition of recombinant human bone-morphogenetic protein-2 (rBMP-2) to reconstituted bovine collagen in the product InFuse™ in 2002 for spine procedures. Finally, in 2005, platelet-derived growth factor (PDGF)-1 delivered with a tricalcium phosphate crystal was approved for periodontal regenerative procedures (Gem 21S™). InFuse™ was subsequently approved for long-bone and oral surgical procedures in 2004 and 2007, respectively.

Of the 3 drug-eluting bone graft products that have been FDA approved, efficacy of each has been in question either prior to approval (OP-1™ and Gem 21S™) or post-approval (InFuse™). Further, InFuse™, which was designed to deliver high doses of BMP-2, has been reported to have serious life-threatening side effects, and is exceptionally expensive to use. Despite these risks and limited predictability, the market for InFuse™ is approaching $1 billion, demonstrating the demand from clinicians in orthopedics and oral surgery for a new and suitable synthetic bone graft substitute.

Many growth factors, such as BMP-2, have been shown to interact with long chains of sulfated disacharrides known as heparan sulfate (HS) glycosaminoglycans (GAGs), which are found on matrix and cell-surface proteoglycans throughout the body [1-5]. HS GAGs regulate cell function by serving as a co-factor, or co-receptor in GF interactions with their receptors, and HS GAGs are necessary to induce physiological signaling and growth factor activity [1-7]. Much evidence has accumulated to show that GF signaling is in fact controlled by HS, even in the osteogenic lineage [8-11] or in chondrogenic differentiation [12,13]. The interaction of BMPs with their threonine-serine kinase BMP receptors is also thought to be mediated by HS [13-16]. Also, HS is thought to prevent diffusion of growth factors, such as the BMPs, away from the regions where they are likely to be required [17], and the concept of HSPG as a bioactive vehicle for GF delivery has been substantiated [12,13,18,19]. Highly sulfated forms of the proteoglycan GAG chondroitin sulfate (CS) have also been implicated in GF co-activation [20-23], though these interactions are less well characterized.

Unlike recombinant proteins, HS and CS GAGs are quite heterogenous due, in large part, to post-translational addition, then removal, of sulfate groups to various positions on the constituent monosaccharides, orchestrated by a battery of enzymes in a tissue-, or cell-specific manner [24]. Following addition of a linker sequence at the canononical SGD sequence, variability begins with initiation of a glycosaminoglycan polymer, followed by chain elongation to varying lengths with varying degrees of isomerization, deacetylation, and sulfation followed by desulfation throughout the chain [24]. We have, therefore, investigated whether it would be feasible to deliver the DNA pro-drug to generate a soluble PG *in situ* that would augment the activity of growth-factors, including BMP-2, *in vivo*.

We report here on recombinant human perlecan domain 1 (rhPln.D1) binding and enhancing the activity rhBMP-2 *in vitro*, and we report on the *in vivo* dose-enhancement of rhBMP-2 by the *pln.D1* expression plasmid delivered together as a lyophilized single-phase on a particulate tricalcium phosphate scaffold to generate *de novo* bone formation on the maxillary ridge in a rat model.

## Methods

### Synthesis and ELISA characterization of rhPln.D1

The recombinant proteoglycan rhPln.D1 was generated from HEK 293 cells by an adenoviral expression system originally designed to express the first 247 amino acids of perlecan (initially named HSPG-2), and was characterized as previously described displaying a CS:HS ratio of approximately 2:1 [25]. Additional ELISA characterization of the CS GAG chains was performed by the following methods. The rabbit polyclonal antibody to perlecan (CCN-1) was produced in house against purified human endothelial perlecan. The mouse monoclonal antibody to perlecan domain I (clone A71) was purchased from Thermo Scientific, Rockford, IL, USA. Mouse monoclonal antibodies against heparan sulfate (HS) (clone 10E4), heparinase (Hep) III generated HS stubs (clone 3 G10), chondroitin sulfate (CS) types and D (clone LY111) and CS type D (MO-225) were purchased from Seikagaku Corp., Tokyo, Japan. The monoclonal antibody against CS types A and C (clone CS-56) was purchased from Sigma-Aldrich, St. Louis, MO, USA. Mouse monoclonal antibodies against heparin (clones 2Q546 and A7.10) were purchased from US Biological, Marblehead, MA, USA and Merck Millipore Billerica, MA, USA respectively. Mouse monoclonal antibodies reactive to the linkage regions of chondroitin sulfate stubs that remain after chondroitinase ABC digestion (clones 1B5, 2B6 and 3B3) as well as CS antibody (clone 7D4) were gifts from Prof. Bruce Caterson, Cardiff University, UK. Biotinylated anti-mouse or anti-rabbit whole immunoglobulin (Ig) secondary antibodies and streptavidin-horse radish peroxidase (SA-HRP) were purchased from GE Healthcare, Little Chalfont Buckinghamshire, UK. Horseradish peroxidase (HRP) conjugated goat anti-rabbit IgG antibodies were purchased from Merck Millipore, Billerica, MA, USA. Endoglycosidase enzymes, proteinase free chondroitinase ABC (C’ase ABC) and heparinase III (Hep III) (EC 4.2.2.8), were purchased from Seikagaku Corp., Tokyo, Japan. Samples were digested with 50 mU/ml C’ase ABC in 0.1 M Tris acetate, pH 8, at 37°C for 16 h to determine the presence of CS. Samples were digested with 50 mU/ml Hep III in 10 mM Tris–HCl, pH 7.4, at 37°C for 16 h to determine the presence of HS. Purified Pln 247 (10 μg/ml), with and without endoglycosidase digestion, was coated onto high-binding 96 well ELISA plates for 2 h at RT. Wells were rinsed twice with Dulbecco’s phosphate buffered saline (DPBS), pH 7.4, followed by blocking with 0.1% (w/v) casein in DPBS for 1 h at RT. Wells were rinsed with DPBS containing 1% (v/v) Tween 20 (PBST), followed by incubation with primary antibodies diluted in 0.1% (w/v) casein in DPBS for 2 h at RT. Wells were rinsed with PBST followed by incubation with biotinylated secondary antibodies (1:1000) diluted in 0.1% (w/v) casein in DPBS for 1 h at RT, rinsed with PBST and then incubated with streptavidin-HRP (1:500) for 30 min at RT. Binding of the antibodies to the samples was detected using the colourimetric substrate, 2 mM 2,2′-azino-di-3-ethylbenzthiazoline sulfonic acid (ABTS) in 0.5 M sodium citrate pH 4.6, and absorbance measured at 405 nm.

### Ligand binding assays

Ligand binding assays were performed in polystyrene microtiter wells as a modified enzyme linked immunosorbent assay (ELISA) as previously described [25]. Either rhPln.247 or capture antibody CSI 001–71 (Antibody Shop) were coated overnight at 4°C onto the surfaces in PBS/N_3_ at approximately 1–2 μg/ml. All wells were then washed and blocked in PBS containing 0.1% Tween. Capture antibody was followed by rhPln.247 (1 μg/ml, produced in our laboratory) in sequence, then rhBMP-2 (R&D Systems) or rhBMP-7 (R&D Systems) were serially diluted. Primary biotinylated anti-BMP murine antibodies (US Biologicals) were applied in PBS/Tween at a concentration of 0.5 μg/ml overnight at 4°C. Alkaline phosphatase conjugated secondary antibody (Goat-α-mouse, Abcam) or streptavidin (Abcam) was applied at a concentration of 1 μg/ml for 2 h then AP activity was monitored at 405 nm by hydrolysis of the substrate 4-Nitrophenylphosphate (Sigma) in 10 mM Tris, pH 9.5 with 5 mM MgCl_2_ (ELISA Substrate buffer) using a Bio-Tek Instruments, Inc. μQuant spectrophotometer (absorbance maximum of 3.0 ELISA units). Background BMP binding in the absence of rhPln.247 was subtracted. Apparent dissociation constants (*K*_*d*_) were derived as previously described [26].

### Osteoblast activation assays

Mineralization assays: MC3T3-E1 subclone 4 osteoblasts (ATCC) were seeded to confluence, 2×10^4^/well, in 2 cm^2^ tissue culture treated TPP dishes in Gibco Growth Medium (Invitrogen custom product, MEM Alpha without ascorbic acid). After 48 h, the medium was changed to Mineralization medium (Growth Medium containing 50 μg/ml ascorbic acid, 3 mM NaHPO_4_) with 8 ng/ml rhBMP-2 (R&D Systems). To three pairs of wells was added rhPln.D1 (rhPln.198 produced in our laboratory) to a final concentration of 1, 2, and 4 μg/ml. Cells were cultured for an additional 6 days after which time the cells were washed once in sterile water, fixed for 30 minutes in 10% neutral buffered formalin, washed twice again with water, then stained for 2 minutes in 2% alizarin red. Densitometry was performed after imaging with a Kodak GelLogic 1500 and using Kodak MI software for grid ROIs. Alkaline phosphatase (AP) activity assays: In a protocol similar to ligand binding assays described above, microtiter wells were coated with a solution of 140 nM rhPln.247 overnight, blocked, then a dilution series of rhBMP-2 was captured overnight. In parallel wells, an equivalent amount of BMP-2 (as validated by ELISA) was directly coated in the wells using an identical dilution series. All wells were washed and blocked again with PBS/Tween then washed with PBS and seeded with pre-osteoblasts in alpha-MEM with 10% serum, 2.5x10^4^/well in 100 μl. After a 6 h seeding period, media in all wells was replaced with Mineralization media (after imaging to ensure even seeding). After 5 days incubation, pre-osteoblast activation was determined by AP activity released by pre-osteoblasts into the serum-free medium. To measure relative AP activities, 20 μl conditioned medium from each well was transferred to 80 μl of ELISA Substrate buffer and color development allowed to proceed for 24 h at 22°C. K_act_ for each condition was determined from dilution series equilibrium point representing 50% maximum activity release.

### Bioactivated TCP particle preparation

To prepare bioactivated crystals for surgical implantation in rats, a solution of rhBMP-2 (up to 50 ug per ml of final TCP volume) with plasmid DNA lipoplexes (up to 120 ug DNA per ml of final TCP volume plus lipid at 1:2 v:v DNA:lipid) was made and mixed with TCP particles (Impladent, Inc., 200–600 μm), usually 0.5 ml DNA lipoplex solution for 1 ml of TCP. First, washed TCP particles were loaded with rhBMP-2 and incubated for 20 min at 22°C. Second, the same TCP particles were further bioactivated by adding either *pln.247* plasmid (pln.247 in pBI vector, Clontech) or an empty-vector plasmid in a cationic lipoplex (prepared and incubated for 20 min at 22°C (see DNA lipoplex preparation). The TCP particles and solutions were well mixed and incubated for 1 hr at 22°C. Greater than 90% of the biologics within the loading solution were adsorbed to the TCP particles when loading for *in vivo* application in this manner. The bioactivated TCP particle mix was then frozen at −80°C for 1 hr and lyophilized overnight at −40°C with 100 x10^-3^ mBar pressure. The lyophilized TCP particles carrying both rhBMP-2 and *pln.D1* expression plasmid are designated throughout the article as B-247. To measure biologic adsorption to the TCP, then kinetic release of loaded biologics, excess bioactivation solution was removed from the TCP for biologic concentration determination just prior to freeze-drying. Adsorption and elution were densitometrically determined for BMP-2 by immunoblot analysis and for *pln.D1* plasmid from agarose gel analysis, where biologic concentrations after TCP loading, then elution, were calculated from standard curves.

### DNA lipoplex preparation

Plasmid DNA was mixed with Lipofectamine 2000® (Invitrogen) in a 1:2 v:v ratio in sufficient volume to mix well with the TCP particles. The DNA and lipid suspension was incubated for 20 min at 22°C to allow for complexing. Sucrose was added to the DNA lipoplex solution to a final 2% w/v of the final crystal volume.

### qPCR

**Figure 1:**
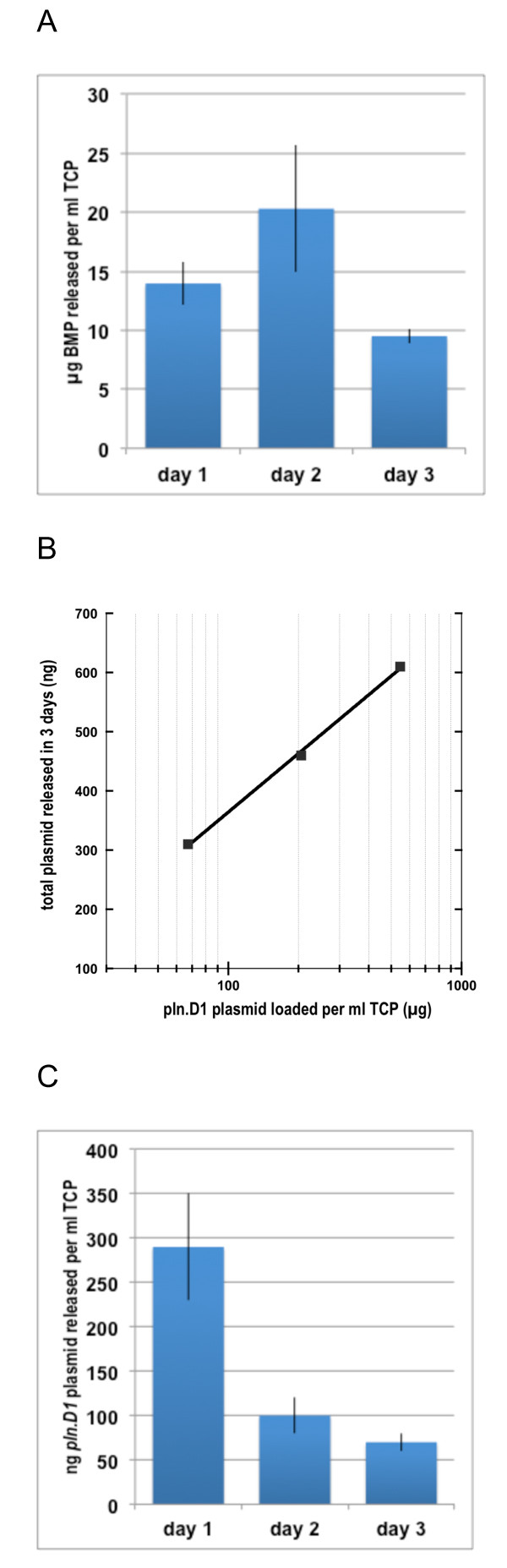
**A: Daily elution of BMP-2 per ml of TCP that had been loaded then lyophilized with BMP-2 amounts ranging from 100 μg to 1 mg.B**: Log-linear relationship between total *pln.247* plasmid eluted after 3 days (y-axis) and *pln.247* plasmid loaded per ml of TCP (x-axis). C: Daily elution of *pln.247* plasmid per ml of TCP that had been loaded then lyophilized with approximately 205 μg *pln.247* plasmid.

qPCR reactions were performed with SYBR Green Master (Sigma) mix using an initial denaturation of 95°C for 3 min then 50 cycles of 95°C for 30 sec and 63°C for 30 sec. The forward primer originated in the 5′ untranslated region (5′-AAC tCC gCC CCA ttg ACg CA-3′) and the reverse primer originated within the transgene sequence (5′-gCA tCt ggC tgg Cgg tCA Ct-3′) of the *pln.247-mod* expression plasmid (Figure 1). PCR cDNA was generated from reverse transcription reactions using random primers. Calculated thresholds (Ct) were determined automatically with the maximum curvature approach on a BioRad MyiQ™ qPCR machine.

### Animal care and procedures

All animal studies were performed in compliance with and under the oversight of the Institutional Animal Use and Care Committee of the University of Alabama. Retired breeder male Lewis rats (Charles River Laboratories), >250 g, were maintained in standard housing, two per cage, with bedding changed 3x weekly. Food and water were provided *ad libitum* and replaced as needed. Rats for each procedure were chosen randomly, and studies were performed in a double blind manner so that the operator did not know which rat or location was receiving experimental or control products. Rats were anesthetized with isoflurane (4-5% induction, 2% maintenance) for all procedures. Animals were monitored daily for signs of distress and closely examined if distress was apparent. Animals with infections or other issues were excused from the study and euthanized prior to harvesting the maxilla in accord with methods approved by the AVMA Panel on Euthanasia and with IACUC oversight.

### Bone void surgical procedure

In anesthetized rats and in a sterile field, bilateral full-thickness gingival flaps were raised 2 mm mesial to each maxillary first molar on the edentulous ridge. A 2 mm diameter round bur rotating < 200 rpm with a saline drip was used to create a void crater defect in the bone approximately 2 mm wide and 1 mm deep. Surgical defects were rinsed and wiped clean, then control or experimental graft materials were placed and condensed to the level of the natural bone. A 5 mm diameter GTR barrier membrane was placed onto the bone covering the defect and the flaps were closed and fixed with cyanoacrylate. The treated animals were fed soft chow during the first week post-surgery and healing permited for 2 or 3 weeks.

### Maxillary ridge augmentation

In anesthetized rats, full-thickness tunnel flaps were raised bilaterally on the edentulous maxillary ridges mesial to the first molar. Experimental and control bone grafts were inserted under the flaps onto the bone surface. Tunnel entrances through the gingiva were sealed and healing permitted for various periods of time ranging from 3 days to 12 weeks. The treated animals were fed soft chow during the first week post-surgery.

### CT analysis

Maxilla were harvested as whole units, and fixed in Histochoice (Amresco), for CT analysis. μCT isosurface images were acquired using a 70 kVp-25ma-20 ms protocol and reconstructed at 52 microns resolution with a threshold of 600 voxel units under service contract with Charles River Imaging. Three-dimensional (3-D) isosurface rendered images were generated using an isosurface threshold of 600 voxel. 3-D raw data were imported into image processing software Amira® for segmentation and new bone was segmented manually.

### Histological analysis

After harvesting and fixation, maxilla were prepared for histological analysis by decalcification in 5% formic acid and paraffin embedding. Embedded blocks were step serially cut to produce 7 micron sections for staining and analysis.

## Results

### BMP binding and activation by rhPln.D1 in vitro

**Figure 2:**
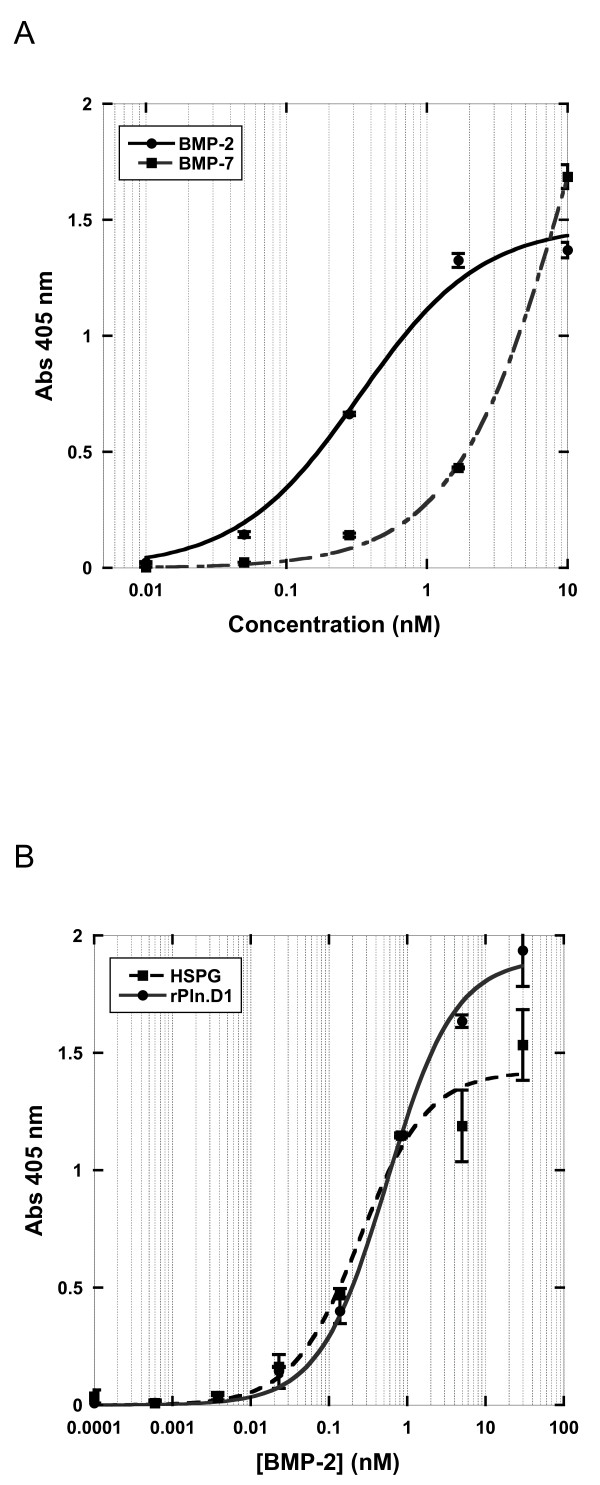
**Ligand Binding Curves; A: rBMP-2 (solid line) and rBMP-7 (dotted line) dilutions were bound in steady state to immobilized rhPln.D1.** Retained BMP was detected by anti-BMP antibodies. Y-axis is Abs 405 nm; X-axis is rhBMP-2 and rhBMP-7 concentration in nM units. Equilibrium point (apparent K_d_) is 0.4 nM for binding of rBMP-2 in this example. **B**: Either rhPln.D1 (circle, solid line) or a sample of HSPG (square, dashed line) were immobilized to bound CS-0071 anti Pln.D1 antibody then bound in steady state to dilutions of rBMP-2. Y-axis is Abs 405 nm; X-axis is rhPln.D1 and HSPG concenration in nM units. In this example, apparent Kd for BMP-2 binding with rhPln.D1 = 600 pM and for BMP-2 binding with HSPG = 150 pM after subtracting background. Error bars represent standard deviations of duplicates.

**Figure 3:**
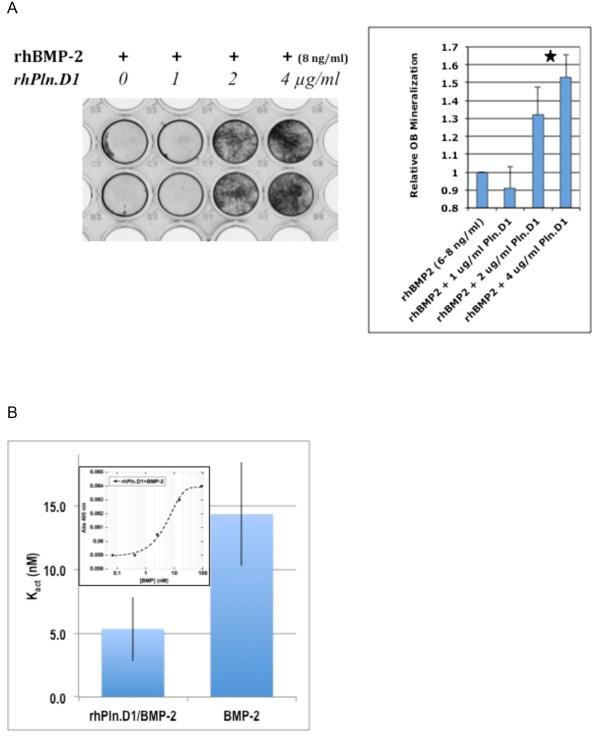
**A. Osteoblast Activation.A**: Mineralization Assay. Left; pre-osteoblasts were seeded evenly to confluence and treated for 6 days with 8 ng/ml rhBMP-2 plus varying concentrations of the rhPln.D1. Dark stain represents dose-dependent mineralization as a result of alizarin red staining. Right; Stained mineralization in the wells was quantified by densitometry. Data represent fold increase beyond the level of mineralization seen with low level BMP-2 alone. Star represents statistical difference between the mineralizing effects of both 2 and 4 μg/ml Pln.D1 conditions with either 0 or 1 μg/ml Pln.D1 (p = 0.02). Data represent replicates in three separate experiments. **B**. Alkaline Phosphatase Assay. Activation of pre-osteoblasts was determined by AP activity released by pre-osteoblasts into serum-free medium after 5 days incubation. Conditions compared BMP-2 dilutions immobilized either on plastic (BMP-2) or onto rhPln.D1-coated wells (rhPln.D1/BMP-2). Equal levels of BMP-2 in wells was confirmed by ELISA (not shown). K_act_ for each condition was determined from dilution series equilibrium point representing 50% maximum activity release (inset).

We have previously shown that the soluble rhPln.D1 expressed from mammalian cells either by adenoviral infection or by plasmid transfection binds to BMP-2 (mature form) and to the following growth factors; BMP-6, BMP-14, FGF-2, VEGF 189, and PDGF-BB as described [25]. The binding of rhBMP-2 to immobilized rhPln.D1 was tight (573 ± 51 pM apparent K_d_), and was approximately 10 fold greater than binding to rhBMP-7 (Figure 2A). For comparison, binding of BMP-2 to a commercial source of HSPG (150 nM) was approximately 4 fold more avid than binding to the rhPln.D1 Figure 2B). The inverse of this ligand binding design, between immobilized BMP-2 and soluble rhPln.D1, demonstrated no measurable binding at all (data not shown). As previously demonstrated for FGF-2, these BMP-2 binding data and apparent K_d_ were similar for two different rhPln.D1 recombinant proteoglycans (rhPln.198 and rhPln.247 (see [25]) (not shown). Functionally in cell culture, pre-osteoblasts were differentiated to produce mineralized substrate by co-delivery of rBMP-2 with the rhPln.D1 proteoglycan *in vitro* (Figure 3A). Using alkaline phosphatase release as an alternative assay for pre-osteoblast differentiation, there was a 2.7 fold enhancement of the BMP-2 dose-effectiveness when rBMP-2 had been captured with immobilized rhPln.D1 compared to the effect of BMP-2 only in these assays, reported as K_act_, (p = 0.03, 2-sided t-test) (Figure 3B).

### GAG characterization of rhPln.D1 CS and HS

**Figure 4:**
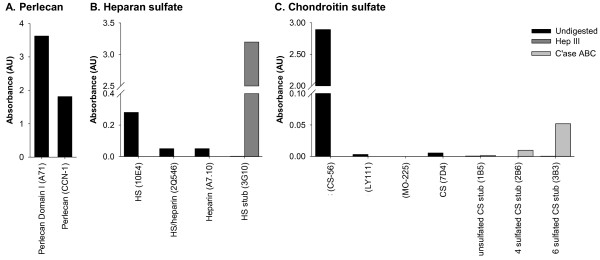
**ELISA characterisation of rPlnD1.A**: Immunoreactivity of protein core using monoclonal anti-perlecan domain I (A71) and polyclonal anti-perlecan antibody (CCN-1); **B**: HS GAG characterization using monoclonal antibodies against HS chains (10E4), HS/heparin (2Q546), heparin (A7.10) and HS stub detection (light bars) after digestion with Hep III (3G10); **C**: CS GAG chain characterization using monoclonal antibodies against CS tetrasaccharide type A-D (CS-56), CS hexasaccharide type C-C-A (LY111), CS hexasaccharide type C-A-D (MO-225), CS (7D4) and CS stubs (1B5 (unsulfated), 2B6 (4S) and 3B3 (6S)) detected after no treatment (dark bars), or chondroitinase ABC digestion (light bars). Data corrected for background absorbance and presented as mean ± standard deviation (n=3).

Additional evidence of a recombinant perlecan core protein was provided by the polyclonal CCN-1 (Figure 4A). The HS of the rhPln.D1 pool was reactive with antibody clone 10E4 indicating the presence of N-sulfated glucosamine residues, but was not reactive with mAb A7.10 or mAb 2Q546, which recognize more highly sulfated, or heparin-like, regions of HS (Figure 4B). Digestion of HS with Hep III exposed an HS stub epitope that was immunoreactive with mAb 3 G10 further confirming the HS GAG character. The CS of the rhPln.D1 pool was reactive with antibody clone CS-56, which reacts with CS types C, D, and A (Figure 4C). However, the recombinant was not reactive with antibody clone LY111 that recognizes a CS type C and type A. Nor was the rhPln.D1 recognized by mAb MO 225 suggesting lack of a reducing end CS-D disaccharride. Upon CS GAG digestion with chondroitinase ABC, there was weak immunoreactivity with mAb 3B3 (anti-GalNAc(6S) stub), and relatively less reactivity with mAb 2B6 (anti-GalNAc(4S) stub) and no reactivity with mAb 1B5 (anti-unsulfated stub).

### Biologic retention and release of pln.D1 plasmid and rBMP-2

Kinetic data demonstrated that the BMP-2 bound efficiently to the TCP particles (at least 2.4 mg rhBMP-2 per ml of dry TCP bound in 2 hours at 4°C). The expression plasmid also adsorbed quickly, up to 240 μg/ml TCP particles in 2 hours at 4°C. After lyophilization of TCP particles that were adsorbed with rhBMP-2 amounts ranging from 100 μg up to 1 mg per ml of TCP, the rhBMP-2 was slowly released into 37°C PBS buffer (43 ± 2 μg/ml of TCP released in the first 3 days), as detected by immunoblot analysis (Figure 1A). The pln.D1 plasmid was also eluted slowly but, unlike with BMP-2, was released in amounts directly proportional to the amount of plasmid loaded (Figure 1B). Over the initial 3 days of elution of TCP augmented with 205 μg of *pln.247* plasmid, approximately 460 ng ± 30 ng plasmid was released at 37°C (11.2%), mostly during the first 24 h (Figure 1C).

Substituting a GFP expression plasmid (EGFP-pcDNA3.1+) for the *pln.247* plasmid *in vitro*, the loaded and lyophilized crystals, when placed with HEK cells in culture wells, delivered biologically active plasmid to the cells, evident by fluorescent protein expression as early as 16 h incubation (not shown).

### In vivo rhPln.D1 expression

**Figure 5:**
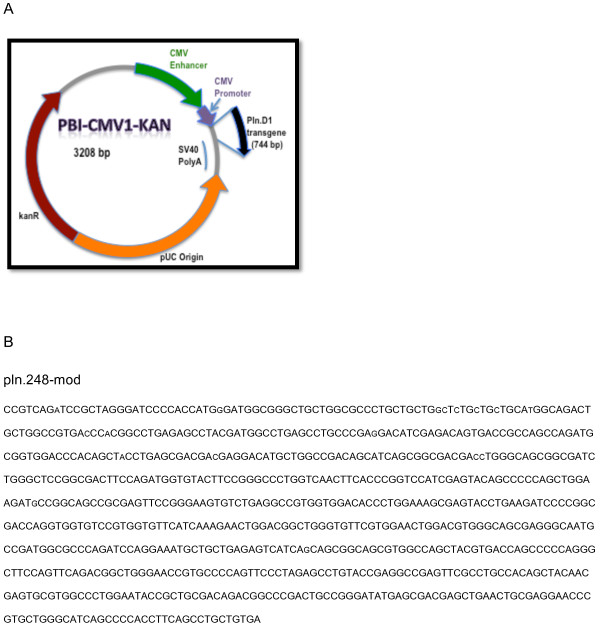
**A: Plasmid map of the codon-modified perlecan D1 expression construct used for *in vivo *expression analysis.B**: sequence of the codon-modifed perlecan D1 transgene employed for *in vivo* expression analysis.

PCR of biopsies taken up to 12 weeks after B-247 implantation demonstrated the presence of retained plasmid (data not shown). To specifically measure *in vivo* expression of perlecan RNA from the bioactivated TCP particles, a codon-modified pln.D1 expression plasmid was used (*pln.248-mod*, Figure 5A and B) allowing discrimination of recombinant *pln.D1* mRNA from native. TCP particles loaded with 150 μg/ml of *pln.248-mod* expression plasmid and 75 μg/ml rhBMP-2 were implanted subperiosteally on the bone of the rat maxilla, and after three days, the graft site was biopsied. Agarose gel analysis of PCR using primers specific for the codon-modified expressed transgene and 63°C annealing (not shown), as well as qPCR using 63°C annealing reactions, demonstrated a mean Ct of 21.4 ± 1.7 and a 64 ± 5 fold increased level of codon-modified *pln.D1* mRNA at the experimental site relative to the contralateral control site (p = 0.0005, one-side t-test of Ct, normalized by cyclophilin cDNA (Ct) levels).

### Bone void healing in the rat maxilla

**Figure 6:**
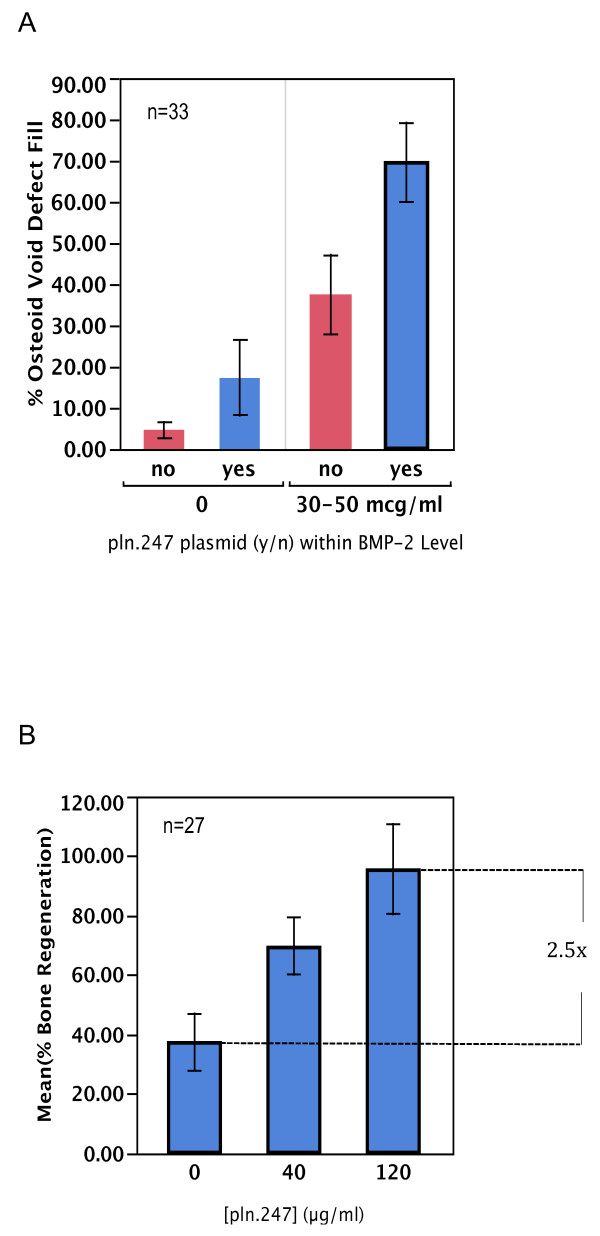
**Histomorphometric subject data of % osteoid fill after 3 weeks healing in a bone void model.A**: wounds treated with TCP particles loaded with (yes) or without (no) 40 μg/ml *pln.247* plasmid in the presence (30-50 μg/ml) or absence (0) of rhBMP-2. **B**: wounds treated with 30-50 μg/ml BMP-2 and varying doses of *pln.247* plasmid. Error bars represent standard deviations of 27 sites.

A series of histomorphometric data were first obtained by measuring osteoid generation in maxillary bone wounds created with a 2 mm rotating bur. In these bone void defects, TCP particles alone generated less than 5% new osteoid after 3 weeks healing (Figure 6A, no plasmid, 0 μg/ml BMP-2). Addition of the *pln.247* plasmid (40 μg/ml) to the TCP (0, yes) generated similarly low levels of osteoid fill, not significantly different than the empty TCP particles. Combining low levels of rhBMP-2 with the TCP particles resulted in a mean defect fill of 40% while adding low levels of the *pln.247* plasmid with the rhBMP-2 resulted in almost twice as much osteoid fill (70%) during three weeks of healing, suggesting that *pln.247* plasmid augmented BMP-2 osteogenic activity in bone wound repair. The augmentation of rhBMP-2 activity in void repair was directly dependent on the dose of *pln.247* plasmid co-presented with the rhBMP-2 (Figure 6B, p = 0.0017, one-way ANOVA). The commitment to bone formation began in the first week and osteoid was forming by day 14 (not shown).

### Maxillary Ridge Augmentation

**Figure 7:**
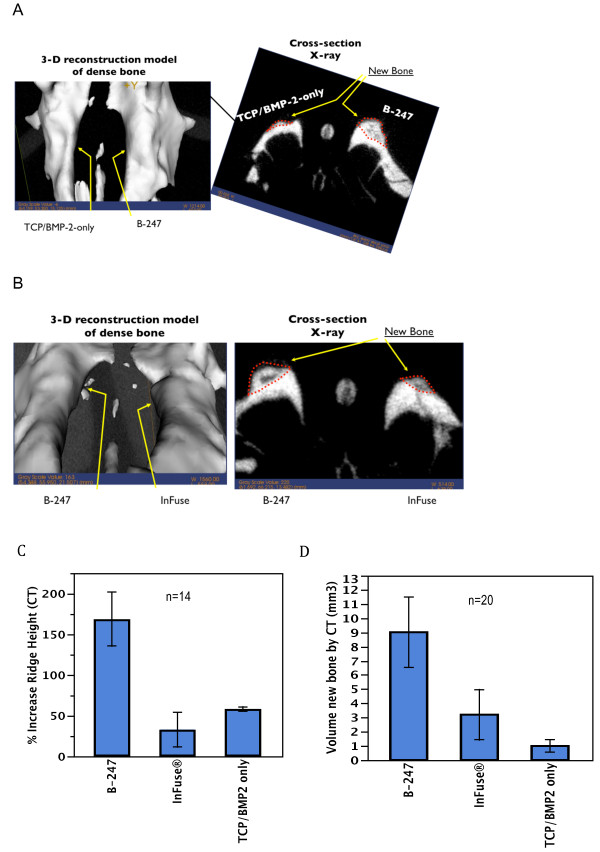
**A and B: μCT isosurface images (left) and cross-sectional radiographs (right) acquired after 12 weeks of healing using a 70kVp-25ma-20ms protocol and reconstructed at 52 microns resolution with a threshold of 600 voxel units.** Dotted lines outline regions of new bone formation. **A**: Left ridge treatment - TCP loaded and lyophilized with 100 μg/ml of rhBMP-2. Right ridge treatment – B-247 treatment of TCP loaded and lyophilized with 100 μg/ml rhBMP-2 plus 100 μg/ml of *pln.247* plasmid. **B**: Left ridge treatment – B-247 treatment of TCP loaded and lyophilized with 50 μg/ml rhBMP-2 plus 100 μg/ml of *pln.247* plasmid. Right ridge treatment – InFuse™. **C**: Percent increase in maxillary ridge hight above normal (100%) after 6 to 12 weeks healing measured in μCT analysis for varying treatment conditions (n = 14). **D**: Volume of new bone assessed by μCT analysis for three treatment conditions after 6 to 12 weeks healing measured in μCT analysis (n = 20).

Experimental and control bone grafts were inserted directly on the crest of the maxillary ridge under full-thickness flaps and healing was permitted for various periods of time. Micro-CT analysis was performed on extracted maxilla after 6–12 weeks healing (Figure 7A and B). By micro-CT, analysis (voxel threshold >600), the B-247 formula (169% ± 33%) was 2.9 fold more effective in building new ridge height than the TCP/BMP-2 only formula (58% ± 2.7%; p = 0.0051) and 5.1 fold more effective than InFuse™ (33% ± 21%; p = 0.0019) (Figure 7C). By micro-CT analysis after 6–12 weeks healing, the volume of new bone generated by B-247 (9.0 ± 2.5 mm^3^) was 9.0 times greater than new bone generated by the TCP particles with BMP-2 only (1.0 ± 0.4 mm^3^; p = 0.02). The B-247 particles also generated 2.8 fold more new bone than the InFuse™ (3.2 ± 1.8 mm^3^; p = 0.05, one-way ANOVA) (Figure 7D).

The mean density of the new bone volume (including marrow spaces) generated in 6–12 weeks by B-247 was not significantly more dense than new bone generated by the TCP/BMP-2 formula but was 4.3 fold more dense than the new bone generated by InFuse™ (33 ± 20; p = 0.008, one-way ANOVA) (not shown).

### Histological analysis

**Figure 8:**
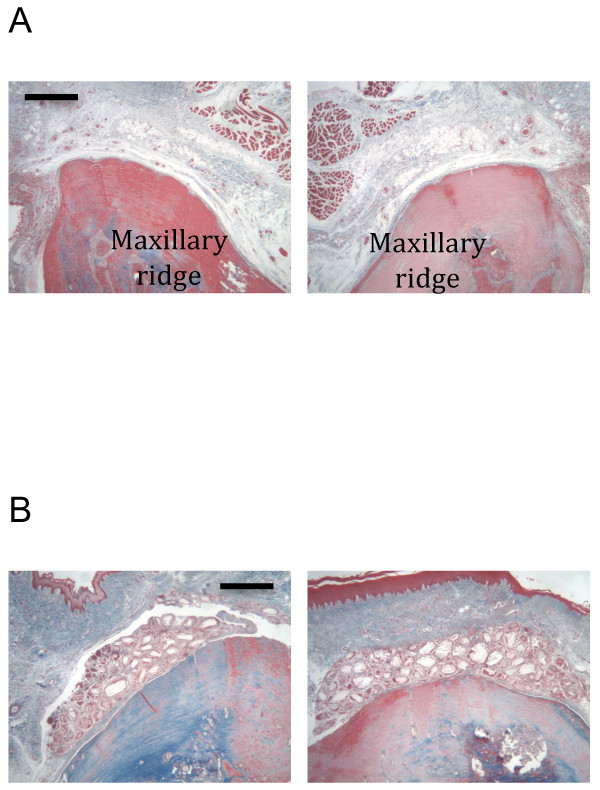
**Masson’s trichrome staining.A**: Untreated maxillary ridge, right and left. Bar = 0.5 mm. **B**: 3 weeks healing. TCP particles only. Bar = 0.5 mm. No new osteoid evident. Pairs of images are from same animal.

**Figure 9:**
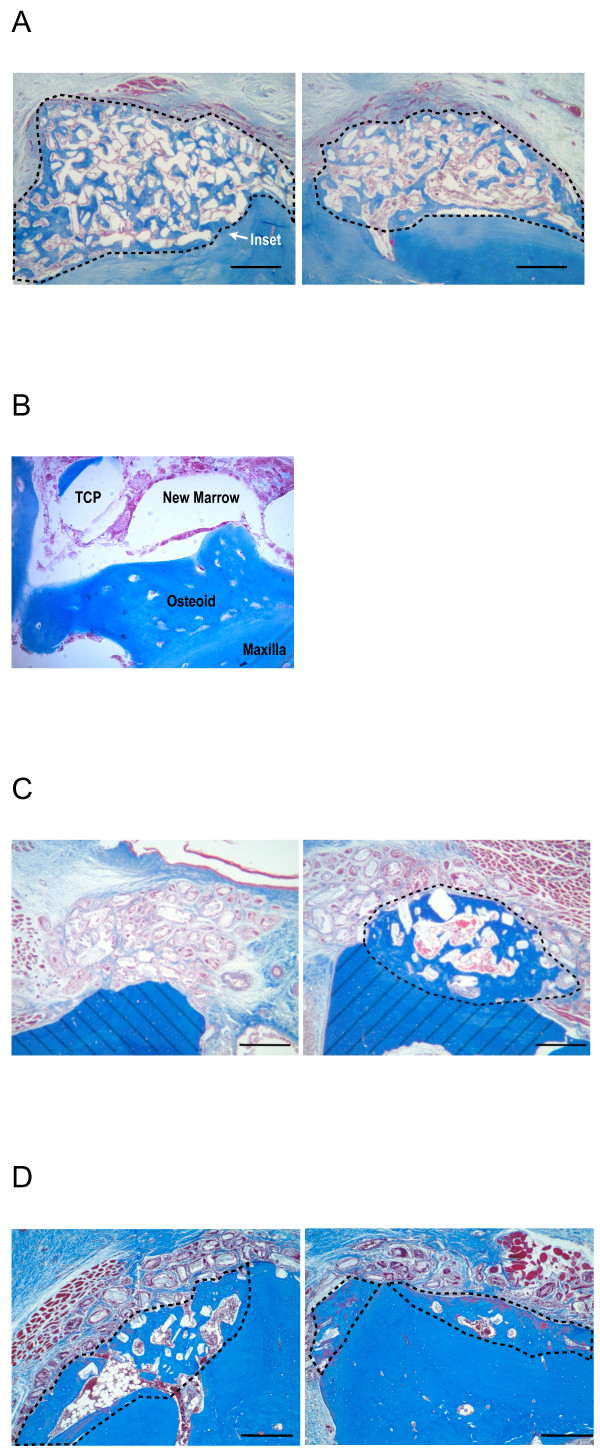
**Masson’s trichrome stain.A**: 3 wk healing after both right and left sides received B-247 treatment of TCP loaded then lyophilized with 50 μg/ml rhBMP-2 plus 100 μg/ml of *pln.247* plasmid. Inset arrow designates region of panel **B**. **B**: 400x magnification of region designated in panel **A** between existing maxilla (bottom) and new osteoid with marrow space and partially integrated TCP. A line of demarcation can be discerned between the labels Osteoid and Maxilla. **C**: 6 wk healing; Left - B-247 treatment of TCP loaded and lyophilized with 50 μg/ml rhBMP-2 plus 100 μg/ml of *pln.247* plasmid. Right - TCP loaded and lyophilized with 50 μg/ml BMP-2. No new bone was generated by the TCP particles activated with only the low-dose BMP-2 (left). Hashed regions represent the original maxillary ridge. **C**: 6 wk healing; Left - TCP loaded and lyophilized with 50 μg/ml rhBMP-2 plus 100 μg/ml of the empty-vector plasmid pBI. Right - TCP loaded and lyophilized with 50 μg/ml BMP-2. Area encircled represents new bone (blue) generated by the bioactivated TCP particles. Bar = 0.5 mm. Pairs of images for **B** and **C** are from same animal. Bar = 0.5 mm.

By histological analysis, the TCP particles, not loaded with any biologic, had no evidence of osteogenesis associated with them in this model of *de novo* bone generation (Figure 8B). In contrast, the B-247 formula of low-dose BMP-2 with *pln.247* plasmid on TCP particles was osteogenic by 3 weeks (Figure 9A, both right and left) clearly developing a non-cartilagenous, intramembranous osteoid network that was integrated into the crest of the maxilla (Figure 9B), was not inflamed, and showed no osteoid of an ectopic nature. By 6 weeks, the new bone generated by the B-247 particles (Figure 9C, right) had begun to mature showing evidence of a central marrow space, while BMP-2-loaded TCP (Figure 9C, left) was not osteogenic in this model, even after 6 weeks incubation. Delivery of low-dose BMP-2 plus the empty-vector plasmid on the TCP particles as a control demonstrated moderate osteogenic activity at 6 weeks healing (Figure 9D, left), slightly more than the BMP-2/TCP combination in this animal (Figure 9D, right).

**Figure 10:**
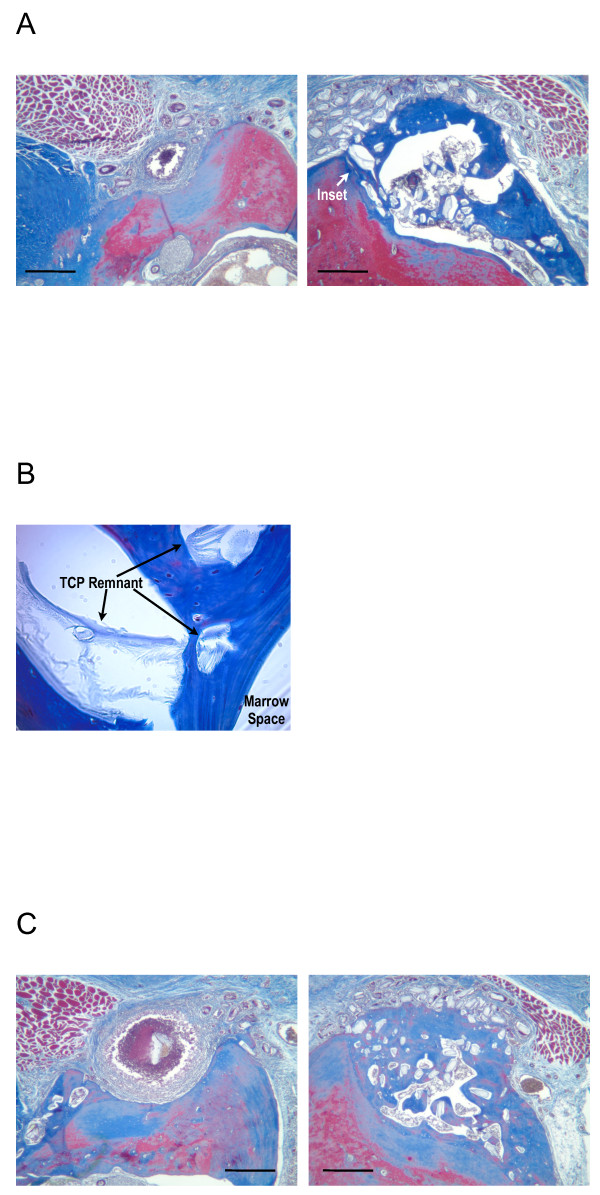
**Masson’s Trichrome stain, 12 wk healing.A**: Left - TCP loaded and lyophilized with 50 μg/ml BMP-2. Right - B-247 treatment of TCP loaded and lyophilized with 50 μg/ml rhBMP-2 plus 100 μg/ml of *pln.247* plasmid. Inset arrow designates region of panel B. **B**: 400x magnification of region designated in panel **A** between crest of ridge (towards left) and marrow space. Arrows designate remnant TCP particles which remain partially integrated with new bone (blue) after 12 weeks. **C**: Left - TCP loaded and lyophilized with 50 μg/ml BMP-2. Right - B-247 treatment of TCP loaded and lyophilized with 50 μg/ml rhBMP-2 plus 100 μg/ml of *pln.247* plasmid. Pairs of images are from the same animal. Bar = 0.5 mm.

After 12 weeks, the B-247 particles were still associated with the development of mature bone on top of the existing maxillary ridge (Figure 10A and C, right), and partially integrated with the new bone (Figure 10B). Remnants of TCP particles remained embedded within the new bone. Evidence of a developed foreign body reaction was often seen in association with TCP/BMP-2 only condition (Figure 10A, 50 μg/ml BMP-2; Figure 10B, 100 μg/ml BMP-2).

**Figure 11:**
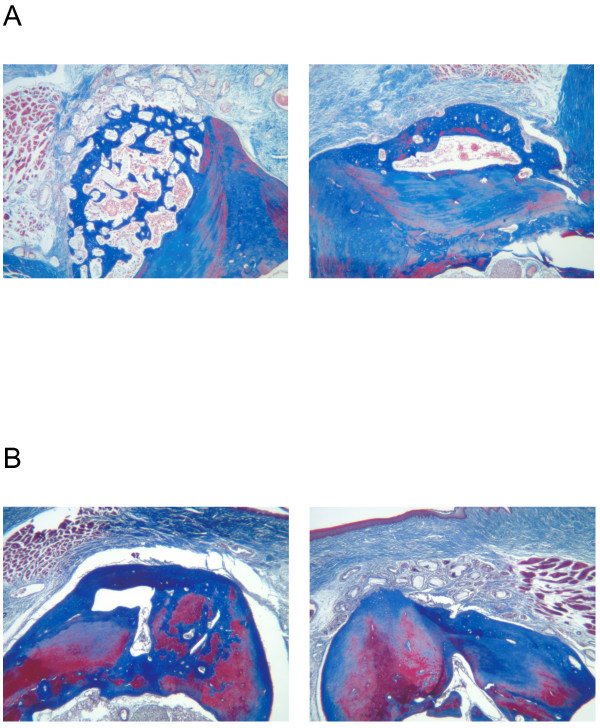
**Masson’s Trichrome stain.A**: 6 wk healing. Left - B-247 treatment of TCP loaded and lyophilized with 50 μg/ml rhBMP-2 plus 100 μg/ml of *pln.247* plasmid. Right: InFuse™. **B**: 12 wk healing. Left - InFuse™; Right - B-247 treatment of TCP loaded and lyophilized with 50 μg/ml rhBMP-2 plus 100 μg/ml of *pln.247* plasmid. Pairs of images are from same animal.

BMP-2 delivered on a collagen sponge (InFuse™) resulted in a loss of maxillary ridge height up to 3 weeks incubation (not shown), but subsequent recovery generated moderate amounts of new bone by 6 weeks (Figure 11A, right), and 12 weeks (Figure 11B, left). The B-247 particles consistently generated more new bone than did InFuse™ (Figure 11A), while InFuse™ was typically more effective by 6–12 weeks than BMP-2/TCP only (Figure 11B).

**Figure 12:**
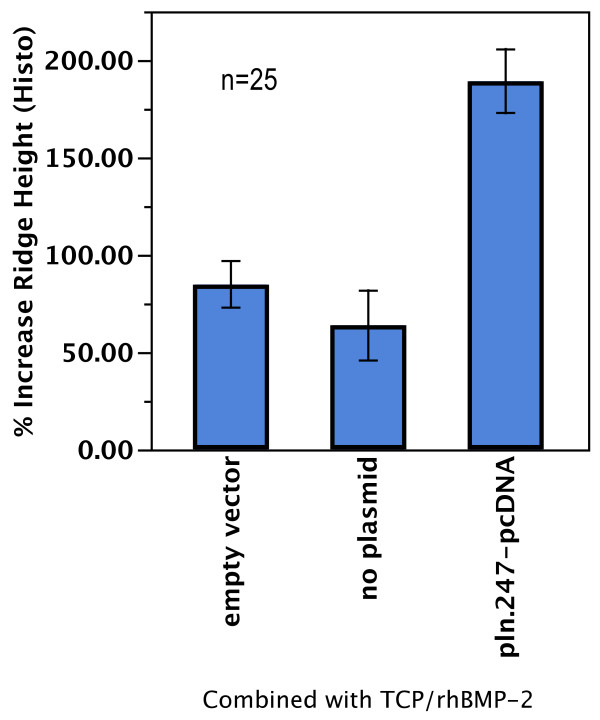
**Histomorphometric summary of conditons that included a BMP-2 dose of 50 μg per ml TCP volume (n = 25).** Measured was % increase in maxillary ridge height (y-axis) relative to the conditions where BMP-2 was combined with either empty-vector plasmid, no plasmid, or the *pln.247* plasmid.

In sites treated with BMP-2 doses with 50 μg/ml of TCP volume (n = 25, Figure 12), the effect of the *pln.247* plasmid on BMP-2 osteogenic ridge height increase measured histomorphometrically was statistically and clinically greater than the BMP-2 only (2.9 fold increase in ridge height, p < 0.0001) and the empty-vector effect (2.2 fold increase in ridge height, p < 0.0025) with no significant difference between treatment with BMP-2 only or BMP-2 plus the empty-vector plasmid (p = 0.46). InFuse™ treatment resulted in a mean ridge height increase of 27% ± 31, significantly lower than the B-247 formulation (p < 0.0001, data not shown).

The CBC and clinical chemistry safety data showed values within normal limits for aging rats. The animals showed no signs of toxicity or general morbidity.

## Discussion

Recent work on HS demonstrated its inherent bent shape and flexibility in solution emanating primarily from the N-acetylated and less highly sulfated regions of the GAG polymer [27]. While overall flexibility of the GAG generally correlated with length, flexibility may be limited regionally. Analysis of the interactions of HS-like polymers with growth factors has demonstrated an intimate interaction between the two, requiring multiple contacts in sulfated regions [28-30]. Similar binding complexity between HSPGs and BMP-2 is expected [16] and such could be implied from the high binding avidity measured here.

Antibody characterization of the *in vitro* recombinant with the anti-HS antibodies suggested this rhPln.D1 HS is N-sulfated, within regions of N-acetylation [31,32], but also that additional sulfation may be limited, as suggested by the lack of interaction with the two heparin-binding mAbs. Further, a proportion of the rhPln.D1 was shown to be expressed with CS, which was immunoreactive with mAb CS-56 [25], demonstrating the presence of sulfated regions of either type C, D, or A. While CS-56 prefers the octasaccharide sequence of CS-type C-A-D-C or A-A-D-C, additional antibody characterization of the rhPln.D1 GAGs demonstrated no immunoreactivity with MO 225 which also prefers the octasaccharide sequence of CS-type C-A-D-C but reacts only weakly with the sequence A-A-D-C [33,34]. No immunoreactivity by mAb LY111, which is an antibody thought to prefer the CS-type C-C-A hexasaccharide without the CS-D disaccharride [33], supports the presence of the disulfated CS-D unit, possibly in the CS-A-A-D-C octasaccharride configuration. Previous characterization suggested a similarity in the rhPln.D1 generated *in vitro* from HEK 293 cells and from primary endothelial cells [25] but differences from this GAG characterization expected with *in vivo* expression remains unclear.

While there is little evidence to support BMP-2 binding of CS, BMP-4 has been shown to interact functionally with a highly sulfated CS [22] suggesting that both the sulfated HS and CS of the rhPln.D1 core used here could be involved in functional BMP-2 interactions.

While the rhBMP-2 bound tightly to rhPln.D1 that had been immobilized directly in solid phase ELISA, immobilizing the BMP-2 directly to the polystyrene substrate prevented the interaction with soluble rhPln.D1. These data could be interpreted to support the requirement that multiple contacts exist between the BMP-2 ligand and sulfated GAG chains that was discussed above. Direct immobilization of the growth factor to a substrate in a ligand binding assay may limit the freedom of the GAG chains to optimally bind the GF ligand, likely through steric hindrance by the immobilization surface.

Binding avidity between the rhPln.D1 and BMP-2 was found to be significantly tighter than binding to BMP-7. This was not expected because both BMP-2 and BMP-7 primary sequences each contain three conserved triplets of basic amino acids in the N-terminal region near the start of the conserved cysteine knot. The two N-terminal triplets of BMP-7 are, however, more distant from the knot than in BMP-2 and contain one less basic amino acid substituted by Ser [16]. Further, newly reported molecular docking analysis of heparin/HS with BMP-2 demonstrates that non-conserved residues provide additional electropositive binding potential compared to BMP-7 [35].

Calcium-based ceramic materials, such as hydroxyapatite and tricalcium phosphate, are effective as osteoconductive agents and work well alone as bone void fillers but they are not osteoinductive (as demonstrated above). Tricalcium phosphate has been shown to be a good delivery vehicle for BMP-2, though, and the combination is osteogenic [36-39]. While very high amounts of rhBMP-2 could be adsorbed to the porous, unsintered calcium phosphate apatite used in these experiments, release of the BMP-2 appeared to be rate limiting. Incubating lyophilized particles that had been loaded with varying BMP-2 amounts ranging from 100 μg to 1 mg per ml of TCP yielded similar quantities of BMP-2 released into solution over the first three days, unlike the dose-dependent elution of the *pln.247* plasmid that was demonstrated. The fact that BMP-2 release was most rapid during the second day of incubation and decreased thereafter suggested that a TCP volume-dependent pool of “early-release” rhBMP-2 had become established during the loading and lyophilization process. Similar loading and release characteristics were obtained from a non-porous unsintered calcium phosphate apatite although released amounts were several fold higher (data not shown), suggesting that the porous TCP structure utilized in these experiments aided in retention of BMP-2. These kinetics of early and controlled BMP-2 release may have contributed to the efficacy and safety of the technology.

To substantiate specific pro-drug activity of the *pln.247* plasmid, we have demonstrated the presence of expression plasmid throughout a 12-week healing period by PCR, and specific mRNA expression of the recombinant core within the wound. However, we have not yet characterized the expressed recombinant within the wound as neither the core nor the associated GAGs can be distinguished from those of native perlecan D1 or other native GAGs with existing tools. These data, therefore, have only indirectly implicated the *in vivo* role of the recombinant HS or CS in BMP-2 dose-enhancement. While the GAG characteristics of the rhPln.D1 used here were shown to be similar whether expressed from HEK 293 cells or HUVEC [25], the type and character of GAG can probably be influenced by the cell type and the environment in which it is expressed [40], although this has not been systematically described in a recombinant system yet. Nonetheless, an analysis of the structure/function relationship, both *in vitro* and *in vivo*, is important to pursue.

The B-247 bioactivated TCP appeared to stimulate intramembranous bone formation that was juxta-proximal to the particles, (see [41]), and new bone that was contiguous with the maxilla. There was no evidence of ectopic bone in this model; the B-247 particles were associated with the development of mature woven bone on top of, and integrated with, the maxillary bone. However, not all B-247 particles were associated with new bone development; in almost every case the most peripheral particles surrounding the developing new bone remained unassociated with new bone formation. This observation might be partly attributable to a natural physiological limitation to volume-increase of the maxillary ridge and a physiologically excessive volume of particles administered throughout the trials. Alternatively, the process of *de novo* bone formation around the particles is expansive resulting in less particle density within the new bone while pushing the remaining unincorporated particles peripherally. It is likely that a combination of these two phenomena resulted in a border of some unincorporated particles. The relative proportion of unincorporated particles was ultimately a function of the osteogenic capacity of the treatment condition – non-osteogenic TCP conditions resulted in up to 100% unincorporation of particles into new bone, while the most osteogenic conditions resulted in the majority of particles being incorporated into new bone. Limitation of new bone to regions of bioactivated TCP deposition might also be interpreted as a function of the highly retentive nature of the particles for the biologics, in part.

The *pln.247* plasmid delivered alone on the tricalcium phosphate particles was not osteogenic. Further, neither non-demineralized human allograft nor very low doses of rhBMP-2 on the tricalcium phosphate particles (<30 μg/ml) were osteogenic with or without the *pln.247* plasmid (data not shown). It was apparent, therefore, that the *pln.247* plasmid was not osteogenic when delivered alone, though not surprising since osteogenesis directly attributable to augmenting only HS/CS proteoglycan levels has never been reported.

The model using retired breeders is not common and was chosen with reason. Declining stem cell populations and decreased healing potential in these older animals would theoretically limit the rate and extent of bone regeneration as compared to what could be expected in young mammals. Given that these data have direct bearing on repair or augmentation of the jaw, and the majority of those needing oral or periodontal reconstruction are not young, the use of older animals was deliberate so as to challenge the technology as it may eventually be challenged in the clinic.

A body of evidence exists in the literature demonstrating a role for HSPGs, and perlecan in particular, in cell adhesion, cell proliferation, cell differentiation, and cell migration, all of which are crucial to osteoinduction and wound repair. As cited herein, CSPGs can also have a similar role. Despite this existing evidence for HSPGs and CSPGs as potentially valuable adjuncts for a variety of tissue engineering and wound-healing strategies that are mediated through growth factor activities, no HS or CS augmented therapies yet exist.

By therapeutically delivering the cDNA encoding the proteoglycan core sequence, this technology may overcome complications in HSPG or CSPG manufacturing with pharmaceutical consistency. The technology allows site-specificity and tissue specificity of the post-translational HS or CS GAG modifications that would be difficult to predict and provide pharmaceutically. The FDA has designated the B-247 TCP particles as a combination product, under the regulation of CBER, which is a first for bone graft devices, as all others are currently regulated as devices in CDRH.

## Conclusions

A recombinant perlecan domain 1 expressed by the *pln.247* plasmid bound rhBMP-2 tightly and enhanced BMP-2 activity significantly *in vitro*. The *pln.247* plasmid more than quadrupled (4–9 fold) the dose-effectiveness of BMP-2 osteogenic activity for *in vivo de novo* bone generation, and the B-247 formula of TCP, BMP-2, and *pln.247* plasmid was greatly superior in efficacy to TCP and other bone graft products. The B-247 provided more than 5 times as much maxillary ridge augmentation than InFuse™ using ≈ 1/30th of the BMP-2 dose, and the B-247 formula generated new bone with 4 fold greater density than new bone generated by InFuse™. The B-247 formula generated minimal inflammation and did not cause osteonecrosis in the first 3 weeks post-implant as did InFuse™. The B-247 formula only generated new bone that was contiguous with the existing bone and only where the implant material was placed. It remains to be seen whether HS/CS proteoglycan technology for BMP-2 activation will be effective in higher mammals and further research is warranted.

## Competing interests

Agenta Biotechnologies, Inc. financed the processing of this manuscript, holds patents relating, in part, to the content of the manuscript describing nucleic acid delivery of perlecan as a therapeutic in mammals, and may benefit indirectly from its publication. Authors DeCarlo, Belousova, Ellis, Petersen, Grenett, Hardigan, and Whitelock have all received fees, funding, stock, stock options, or salary from Agenta Biotechnologies, Inc. There are no other financial or non-financial competing interests relating to the publication of this manuscript.

## Authors’ contributions

AAD oversaw all experiments, performed the bone void fill surgeries, performed the ELISAs, ligand bind assays, and osteoblast activation assays, and wrote the majority of the manuscript. MB made the B-247 and controls for surgery, performed the ridge augmentation surgeries, performed the TCP release experiments and helped with the ELISAs, ligand binding assays and osteoblast activation assays. ALE made the B-247 and controls for surgeries, performed the osteoblast mineralization assays, assisted in the bone void and ridge augmentation surgeries, and assisted in publication of this manuscript. DP assisted in the ridge augmentation surgeries and provided detailed analysis and chemistry of the TCP crystals. HG performed the PCR experiments. PH provided support for experimental design, analyzed data, and helped interpret results. RO analyzed histological data and assisted with data interpretation. ML and JMW performed HS and CS GAG characterization. Histological preparation of the slides was performed under service contract with the University of Alabama at Birmingham Comparative Pathology lab. All authors read and approved the final manuscript.
